# Comparative ecology of Guinea baboons (*Papio papio*)

**DOI:** 10.5194/pb-8-19-2021

**Published:** 2021-05-21

**Authors:** Dietmar Zinner, Matthias Klapproth, Andrea Schell, Lisa Ohrndorf, Desalegn Chala, Jörg U. Ganzhorn, Julia Fischer

**Affiliations:** 1 Cognitive Ethology Laboratory, Germany Primate Center, 37077 Göttingen, Germany; 2 Department of Primate Cognition, Georg-August-Universität Göttingen, 37077 Göttingen, Germany; 3 Leibniz ScienceCampus Primate Cognition, 37077 Göttingen, Germany; 4 Natural History Museum, University of Oslo, P.O. Box 1172, Blindern, 0318 Oslo, Norway; 5 Institute of Zoology, Universität Hamburg, Martin-Luther-King-Platz 3, 20146 Hamburg, Germany

## Abstract

Thorough knowledge of the ecology of a species or population is an essential prerequisite for understanding the impact of ecology on the evolution of their respective social systems. Because of their diversity of social organizations, baboons (*Papio* spp.) are a useful model for comparative studies. Comparative ecological information was missing for Guinea baboons (*Papio papio*), however. Here we provide data on the ecology of Guinea baboons in a comparative analysis on two geographical scales. First, we compare climate variables and land cover among areas of occurrence of all six baboon species. Second, we describe home range size, habitat use, ranging behaviour, and diet from a local population of Guinea baboons ranging near the Centre de Recherche de Primatologie (CRP) Simenti in the Niokolo-Koba National Park, Senegal. Home ranges and daily travel distances at Simenti varied seasonally, yet the seasonal patterns in their daily travel distance did not follow a simple dry vs. rainy season pattern. Chemical food composition falls within the range of other baboon species. Compared to other baboon species, areas occupied by Guinea baboons experience the highest variation in precipitation and the highest seasonality in precipitation. Although the Guinea baboons' multi-level social organization is superficially similar to that of hamadryas baboons (*P. hamadryas*), the ecologies of the two species differ markedly. Most Guinea baboon populations, including the one at Simenti, live in more productive habitats than hamadryas baboons. This difference in the ecology of the two species contradicts a simple evolutionary relation between ecology and social system and suggests that other factors have played an additional role here.

## 1 Introduction

Baboons (genus *Papio*) are widespread across sub-Saharan Africa and the south-western Arabian Peninsula (Anandam et al., 2013). The genus comprises six closely related (phylogenetic) species: chacma baboons (*P. ursinus*), yellow baboons (*P. cynocephalus*), Kinda baboons (*P. kindae*), olive baboons (*P. anubis*), hamadryas baboons (*P. hamadryas*), and Guinea baboons (*P. papio*) (Zinner et al., 2011; Anandam et al., 2013; Walker et al., 2017). Given their wide distribution, the six species occur in a range of different habitats and under various climate conditions (Jolly, 2013). Although baboons are typically associated with savannah and savannah–woodlands, they occupy diverse habitats from deserts (e.g. in Namibia, Mauretania, Niger, Eritrea) to tropical forests (e.g. Guinea-Bissau, eastern Democratic Republic of Congo, western Uganda) and from coastal lowlands to highlands above 3000 m (DeVore and Hall, 1965; Swedell, 2011). Food availability in most baboon habitats is often strongly influenced by fluctuations between dry and rainy seasons (Alberts et al., 2005; Codron et al., 2006; Swedell, 2011). As the broad range of their habitats suggests, baboons occupy a generalist niche and are highly opportunistic omnivores. They eat a wide variety of plant species and parts, arthropods, and occasionally feed on smaller mammals and birds, but at the same time, they may also be very choosy, rendering their diet both catholic and selective (Altmann, 1998; Whiten et al., 1991; Barrett and Henzi, 2008; Swedell, 2011; Anandam et al., 2013).

Although the ecology of baboons is generally well understood, knowledge of their ecology is unevenly distributed among the six species. Whereas South and East African populations of chacma, yellow, olive, and hamadryas baboons have been studied in detail (e.g. DeVore and Hall 1965; Kummer, 1968a; Altmann and Altmann, 1970; Barton et al., 1996; Schreier and Swedell, 2012; Johnson, 2015), comparative data on West African species and populations are scarce (Galat-Luong et al., 2006; Kunz and Linsenmair, 2008; Ross et al., 2011). This research gap concerns in particular Guinea baboons, the westernmost baboon species.

**Figure 1:**
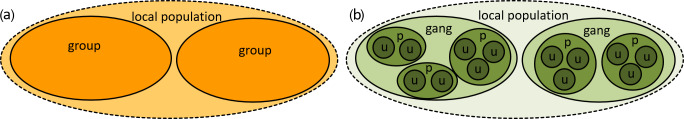
Sketch of the social organization of baboons. **(a)** Uni-level organization of COKY baboons (*Papio ursinus*, *P. anubis*, *P. kindae*, and *P. cynocephalus*). In these species, a group consists of several adult males and females with their offspring. Group sizes can reach more than 100 individuals. Females are predominantly philopatric and form kin-based social networks. **(b)** Multi-level organization of Guinea baboons (*P. papio*) and hamadryas (*P. hamadryas*). At the Centre de Recherche de Primatologie Simenti, units (u) consist of 2–10 individuals (at least one adult male and one to several adult females and their offspring), parties (p) of 30.5 (±6.4), and gangs of more than 60 individuals. The size of our study population at Simenti is 350–400 individuals (>7.5 individuals per square kilometre) and is most likely equivalent to a local population. In hamadryas baboons, corresponding levels of the social organization are one-male unit (OMU), clan, band, and troop (Kummer, 1995).

The social systems (composed of the social organization, social structure, and mating system; Kappeler and van Schaik, 2002) vary among species. Guinea baboons are characterized by female-biased dispersal (Kopp et al., 2015) and share this trait and their multi-level social organization (Fig. 1) with hamadryas baboons, which occur in north-east Africa and the south-western Arabian Peninsula (Kummer, 1968a; Boese, 1975; Sharman, 1981; Hapke et al., 2001; Schreier and Swedell, 2009; Städele et al., 2015; Fischer et al., 2017; Jolly, 2020). In contrast, the other four baboon species (chacma, yellow, olive, and Kinda baboons) live in uni-level social groups (Fig. 1) where female matrilines constitute the core of the groups and males disperse (Swedell, 2011; Jolly, 2020). These four species have been recently dubbed COKY baboons (chacma, olive, Kinda, and yellow) by Jolly (2020). Formerly, these species, together with Guinea baboons, had been referred to as “savannah baboons” – in contrast to the hamadryas or “desert baboon” (Thorington and Groves, 1970; Melnick and Pearl, 1987; Stammbach, 1987). However, the distinction between “savannah” and “desert” baboons does not seem to be justified on ecological grounds, given that, for example, chacma baboons in Namibia or Guinea baboons in Mauretania live in similarly arid habitats as some hamadryas populations in north-east Africa. On taxonomic grounds, the distinction between “savannah” (as *Papio cynocephalus* with four subspecies) and hamadryas baboons (*P. hamadryas*) was also not supported by mitochondrial and nuclear analyses (Zinner et al., 2013; Rogers et al., 2019).

The smallest social unit in hamadryas and Guinea baboons is the one-male unit (OMU or just unit) consisting of one adult male and one to several females and their dependent offspring. Several OMUs form the next level of social organization, a party in Guinea baboons or clan in hamadryas, and several parties (or clans) form a gang in Guinea or a band and hamadryas baboons, respectively (Kummer, 1990; Fischer et al., 2019). Despite the similarity of their social organization, both species differ in other aspects of their social system, their social structure, or social style (Fischer et al., 2019). Guinea baboon males maintain strong social bonds and a high degree of spatial tolerance among each other, and females experience higher degrees of freedom; i.e. they are less restricted in their movements and choice of social partners by their unit male than hamadryas baboon females (Goffe et al., 2016; Fischer et al., 2017).

Attempts to explain interspecific or interpopulation differences in social organization (e.g. group size, numerical sex ratio, sex-biased dispersal) and social structure (e.g. social network, dominance hierarchy) in primates and other species have led to the formulation of the so-called “socio-ecological model” (reviewed in Koenig et al., 2013). This model mainly focussed on ecological factors such as habitat productivity and resource distribution, as well as their impact on the spatial distribution and foraging strategies of females (Crook and Gartlan, 1966; Wrangham, 1979; van Schaik and van Hooff, 1983; Sterck et al., 1997). The distribution of males, in contrast, follows female distribution with the aim of maximizing access to reproductively active females (e.g. Altmann, 1990; Kappeler, 2000).

In baboons, theoretical considerations on the relationships between ecology and social organization have mainly focused on differences between hamadryas and COKY baboons to explain the evolution of their strikingly different social organizations, whereby the social organization of hamadryas baboons was primarily seen as an adaptation to their harsh semi-desert environment (Kummer, 1990; Dunbar, 1988; Barton, 2000).

Although contemporary ecology could only partly explain the variation in primate social organization, other aspects of the social system (e.g. quality of social relationships, levels of aggressiveness, or tolerance) might nevertheless be adaptations to certain ecological conditions (Clutton-Brock and Janson, 2012; Koenig et al., 2013). To analyse and better understand relations between ecological and social variation, data on social systems and respective ecological data of populations and species are needed.

The main aim of our study is to provide basal data on the ecology of Guinea baboons as compared to other baboon species. On a continental scale, we present data of fundamental bioclimatic variables (precipitation, seasonality) and land cover prevalent in the distribution ranges of all six baboon species. These data are the comparative background for our analysis of the ecology of Guinea baboons on a local scale, namely at Simenti in the Niokolo-Koba National Park, Senegal. Furthermore, we contrast some basic aspects of the ecology of Guinea baboons at Simenti with those of hamadryas baboons at Filoha, Ethiopia, and show that the multilevel social system of baboons permits living not only under harsh semi-desert conditions, but also in a variety of different habitats.

## 2 Methods

### 2.1 Continental-scale – interspecific comparison

For the interspecific comparison of climate and land cover characteristics within baboon ranges, we used occurrence data from Chala et al. (2019). Our comparisons are based on 733 presence points: olive baboons 120, yellow baboons 96, hamadryas baboons 64, Kinda baboons 32, Guinea baboons 177, and chacma baboon 244 (Fig. S1). For each species, we calculated averages of two bioclimatic variables (bio 12 annual precipitation (mm a${}^{-1}$) and bio 15 seasonality of precipitation (coefficient of variation = standard deviation of the monthly precipitation estimates expressed as a percentage of the mean of those estimates (i.e. the annual mean)); WorldClim Version2 of ∼1 km resolution; Fick and Hijmans, 2017). We also extracted and compared differences in land cover preference relying on land cover data from the global land cover map for 2009 (Arino et al., 2012).

### 2.2 Local-scale – Guinea baboons at CRP Simenti

#### 2.2.1 Study site

**Figure 2:**
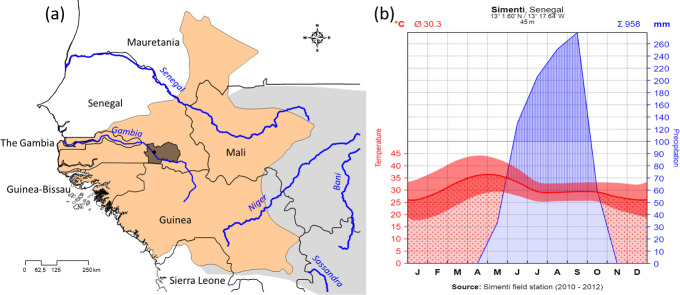
**(a)** Approximate distribution of Guinea baboons (brownish) and position of CRP Simenti within the Niokolo-Koba National Park (PNNK, dark shading). Species distribution after Wallis et al. (2020) and derived from the IUCN spatial database (https://www.iucnredlist.org/resources/spatial-data-download, last access: 19 January 2021). **(b)** Climate graph of the Simenti field site after Walter and Lieth (1967). Depicted are monthly temperatures (red line denotes mean; red band the min, max) and monthly precipitation (blue) in millimetres. Red dotted area demarcates the dry periods (i.e. dry season), the blue area depicts the occurrence of precipitation, and the blue hatched area the humid periods (i.e. rainy season).

We present here basic ecological data from our field site (see Fischer et al., 2017), the Centre de Recherche de Primatologie (CRP) Simenti (13.0262 Latitude, -13.2944 Longitude), in the Niokolo-Koba National Park (PNNK), Senegal (Fig. 2a). The PNNK comprises a variety of different habitat types typical for the Sahelo-Sudanian and Sudanian climatic zone with a pronounced seasonality (Adam, 1971; Arbonnier, 2002; Burgess et al., 2004). The rainy season lasts from June to October (Fig. 2b) with an average annual precipitation of 956 mm in 2010–2012. Our study site lies next to the Gambia River, and multiple seasonal wetlands (Mare) occur in depressions alongside the river. Apart from the riparian forests, prevailing vegetation types are dry forests as well as various savannah types, including savannah woodlands, tree/shrub savannahs, and grass savannahs.

**Figure 3:**
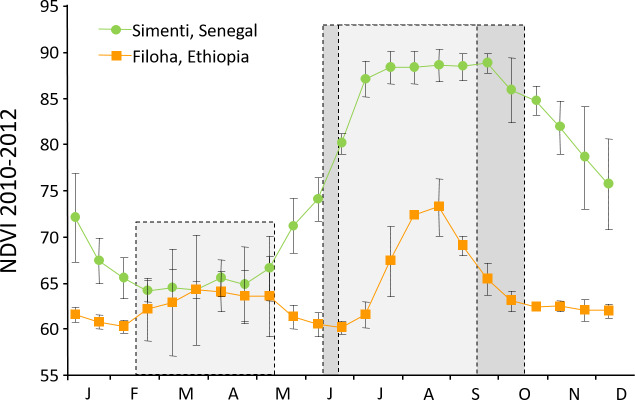
Comparison of monthly normalized difference vegetation indices (NDVI (mean ± SD); measure of “greenness”, i.e. plant productivity) of the Guinea baboon habitat at Simenti, Senegal, and the hamadryas baboon habitat at Filoha, Ethiopia, for 3 years from 2010 to 2012. Since the NDVI can be regarded as an indirect measure for precipitation, the two periods of rain at Filoha (unpredictable short rains from February to May and long rains from June to September, both light grey) and the one in Simenti (June to October, dark grey) are reflected by respective maxima in the graph (source: MODIS NDVI – MOD44/MYD44 (16 d) – TERRA (AM) only; DiMiceli, 2015).

The seasonal climate changes, in particular in precipitation, are followed by seasonal changes in plant productivity as indicated by corresponding monthly normalized difference vegetation indices (NDVIs; Fig. 3). The NDVI is a remotely sensed index of the amount of green plant cover in an area. For comparison, we depicted the NDVIs for Simenti and a hamadryas baboon habitat at Filoha, Ethiopia, for 3 years (2010–2012). Filoha receives one long period of rainfall from late June through September and intermittent and unpredictable short periods of rainfall from February through May (Swedell, 2006). In Simenti, as expected, plant productivity is lowest in the dry season and starts increasing after April and reaches its maximum during the rainy season from July to October. On average, plant productivity is higher in Simenti than in Filoha, even during the dry season.

#### 2.2.2 Habitat classification

**Figure 4:**
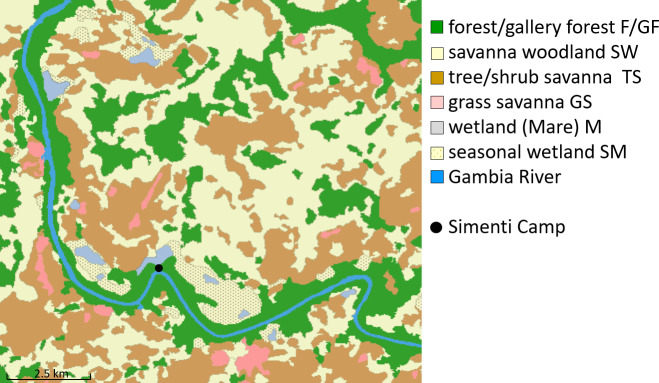
Distribution of habitat classes within the area of interest around CRP Simenti. The black dot indicates the position of the field station of the Centre de Recherche de Primatologie (CRP). We defined the area of interest as the total extent of monitored baboon occurrences in 2010–2012.

We determined six habitat classes according to physiognomic aspects (i.e. structure and appearance) (Klapproth, 2010). These classes are forest, savannah woodland, tree/shrub savannah, grass savannah, temporarily flooded areas, and wetlands. We then employed remote sensing techniques based on multispectral Landsat 5 TM imagery from 28 November 2010 for a supervised habitat classification of the Simenti region. Our area of interest covered 158 km${}^{2}$ and was defined as the total extent of monitored baboon occurrences (i.e. location points enclosed by minimum convex polygons) from 2010–2012 for all monitored baboon parties, given the methods outlined by Johnson (1980). We calculated proportions of habitat classes (Fig. 4) as percentages of the total area of interest.

#### 2.2.3 GPS collars

Our baboon study population at Simenti comprised 5–7 gangs varying in degree of habituation to human observers. Since the ranging patterns of members of the same parties did not differ significantly (Patzelt et al., 2014), we used location data of five individuals representing five parties of three gangs. We repeated this for 3 years so that our spatial analyses were ultimately based on 15 individuals. These individuals were fitted with Tellus Ultra-Light GPS Remote UHF collars. We programmed the GPS devices to take a fix every 2 h during the daytime (seven fixes from 6 to 18 h) and every 3 h during the night (three fixes, 21, 0, and 3 h). Data are available on https://doi.org/10.25625/IHEZUE (last access: 19 January 2021), Zinner et al. (2021). For more information about the capturing and collaring procedures, see Patzelt et al. (2014) and Knauf et al. (2015).

#### 2.2.4 Home range, daily travel distance, habitat use, sleeping sites

For estimates of home range, daily travel distances, and habitat use, we included only daytime fixes, while for sleeping site localizations, we chose one out of three night-time fixes. We applied fixed kernel density estimation (KDE) using the rule-based ad hoc approach (Kie, 2013) to estimate home range (HR) sizes on the 95 % and core area sizes (CA) on the 50 % contour level. For comparison with other studies on baboon home range size, we additionally calculated minimum convex polygon (MCP) HR at the 100 % contours, here defined as the total extent of the area the baboons occupied. To estimate minimum daily travel distance (DTD), we connected consecutive location points for each baboon and summed up the Euclidean distances between points grouped on a daily basis. We only chose days with at least five daytime location points for DTD estimation. We further assessed DTDs on a daily scale to explore possible variations in DTD over the year. We used standard univariate smoothing techniques in a generalized additive mixed model (Fahrmeir et al., 2013) and calculated simultaneous confidence bands at the 99 % level. Based on the supervised habitat map, we estimated habitat use by baboons for the dry and rainy seasons as percentages of location points of individual baboons within respective habitat classes.

#### 2.2.5 Behavioural observations

We followed the baboons on their daily progressions and collected demographic and behavioural data, with a focus on foraging behaviour. We identified woody plant species consumed by the baboons to the species level and noted the parts of the respective plants eaten by the baboons. We then collected examples of corresponding food items for nutritional analyses.

#### 2.2.6 Nutritional analysis

For nutritional analysis, we collected all items (bark, fibre, fruits, nuts, leaves) from plant species eaten by the baboons. We collected samples from those plant individuals that the baboons had been feeding on. Subsequently, we cut the material into slices and stored them on silica gel in an airtight beaker (on average 7 d). We weighed the samples before and after drying and forwarded a dry mass of at least 5.0 g per sample to the Institute of Zoology of the University of Hamburg, where the nutritional analyses were performed. According to the methods used in Bollen et al. (2004), all food items were analysed to the content of nitrogen (reflecting “crude protein”), neutral detergent fibre (NDF), acid detergent fibre (ADF), lipids, sugar (soluble carbohydrates), ash, condensed tannins, phenolics, and alkaloids (only qualitatively in triple assays by reaction with Dragendorff's, Mayer's and Wagner's reagents). The content of the nutrients is presented as percentages per dry mass. Crude protein can be calculated from the nitrogen concentrations by using the formula: crude protein = nitrogen × 6.25 (Maynard and Loosli, 1969). Although commonly used, it usually overestimates protein in plant material, especially in fruit (Conklin-Brittain et al., 1999).

## 3 Results

### 3.1 Continental-scale – interspecific comparison

**Figure 5:**
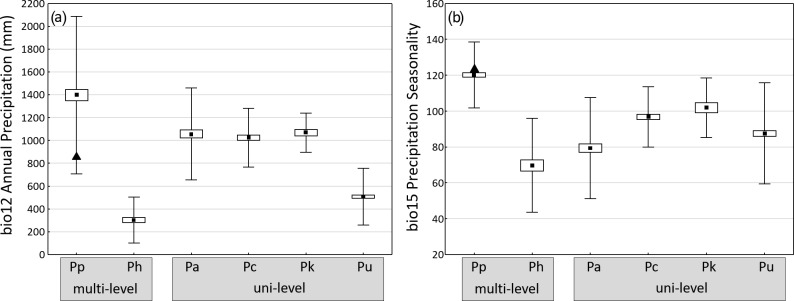
Average annual precipitation **(a)** and coefficient of variance of monthly precipitation (seasonality) **(b)** at occurrence sites of the six baboon species (means ± SE ± SD). Triangles indicate respective values at Simenti (data from WorldClim, variables bio12 annual precipitation and bio15 seasonality of precipitation). Baboon species (number of occurrence sites): multi-level social organization Pp – *P. papio* (177), Ph – *P. hamadryas* (64), uni-level social organization Pa – *P. anubis* (120), Pc – *P. cynocephalus* (96), Pk – *P. kindae* (32), Pu – *P. ursinus* (244).

Guinea baboons have the broadest precipitation range of the six baboon species (Fig. 5a), reflecting the diversity of biomes their range encompasses, from very arid conditions in the Saharan and Sahel region of Mauretania to the wet forests at the coast of Guinea-Bissau, Guinea, and Sierra Leone. Simenti, with precipitation of around 1000 mm per year, lies below the mean. As expected, hamadryas (arid areas at the horn of Africa) and chacma baboons (arid areas of South Africa and Namibia) occur in areas with relatively low precipitation, although chacma baboons also live in reasonably well-watered areas in Zambia, Zimbabwe, and South Africa. Average precipitation values for olive, yellow, and Kinda baboons are similar to the conditions at Simenti.

On average, Guinea baboons occupy areas with the highest average seasonality in rainfall (measured as the variation coefficient of monthly precipitation) (Fig. 5b). The study site lies slightly above the mean, indicating relatively strong seasonal differences. The smallest average seasonal variation is found for hamadryas baboons followed by olive, chacma, yellow, and Kinda baboons. In general, baboons showed overall significant species differences both in average precipitation as well as in precipitation seasonality. Pairwise comparisons indicated the largest difference in both variables exist between Guinea and hamadryas baboons (Fig. 5).

**Figure 6:**
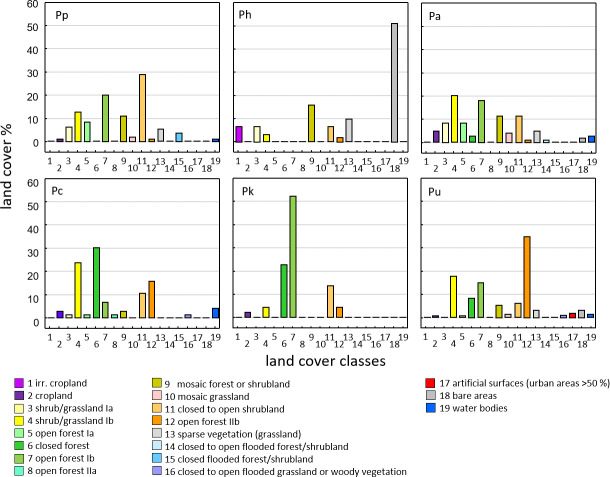
Proportion of land cover classes at sites of the six baboon species (GlobCover, Arino et al., 2012). Baboon species (number of occurrence sites): Pp – *P. papio* (177), Ph – *P. hamadryas* (64), uni-level social organization Pa – *P. anubis* (120), Pc – *P. cynocephalus* (96), Pk – *P. kindae* (32), Pu – *P. ursinus* (244).

Land cover classes found at baboon sites differ among species and reflect the general ecological flexibility of baboons (Fig. 6). For Guinea baboons, classes like open forests or close to open shrubland dominate. As expected for hamadryas baboons, which are often found in semi-desert conditions, bare areas and irrigated cropland comprise large proportions of their range.

### 3.2 Local-scale – the ecology of Simenti baboons

#### 3.2.1 Demography

Between 2012 and 2016, we observed five parties in two gangs and estimated an average party size of around 28 individuals (range 9–40) with 11.4 adults (range 3–21). Since not all juveniles could be identified at the time, numbers of juveniles were only approximated. Variation in adult sex ratio among parties was considerable, ranging from 0.54 to 1.96 (female to male ratio; mean 1.3). The average gang size was 71.2 and 70.4 for the two gangs, respectively. Aside from the observed groups, an unknown number of additional parties/gangs range in the area of interest. A baboon census at the Mare Simenti suggested approx. 300–350 baboons in our study population (Patzelt et al., 2011). For the area of interest, this suggests an estimated population density in the area around Simenti of 7.5–10 baboons per square kilometre.

#### 3.2.2 Home range size, overlap, and daily travel distance

**Figure 7:**
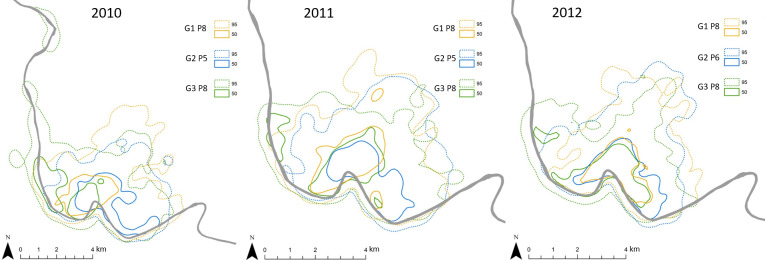
Position, extent, and overlap of annual home ranges (95 %) and core areas (50 %) of Guinea baboon gangs (G) and parties (P). The grey line depicts the Gambia River. Note that the scales vary slightly among years.

**Figure 8:**
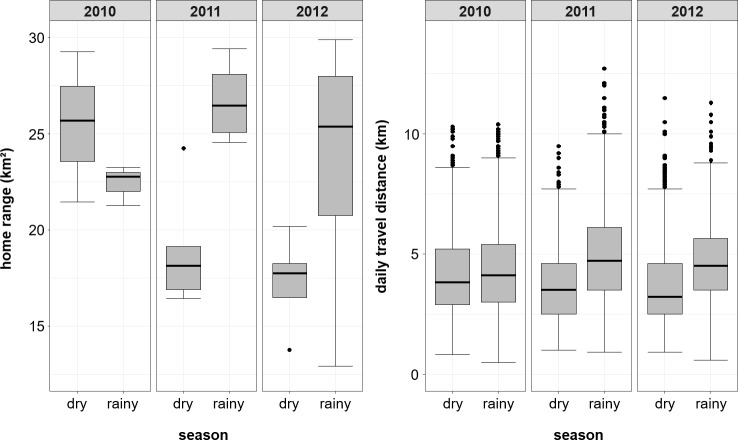
Annual and seasonal variation in home range and daily travel distance (DTD). Boxplots depict the median (black line) and the IQR with the lower (25 %) and upper (75 %) quartile. Boxplot whiskers represent the 1.5 IQR of the lower and upper quartile.

Overall home range size of the Guinea baboons was 24.8 km${}^{2}$ (median, IQR 10.4, $N_{\mathrm{Parties}}=15$) across all years and individuals, with 35.2 km${}^{2}$ (median, IQR 5.3 km${}^{2}$, $N_{\mathrm{Parties}}=5$), 24.8 km${}^{2}$ (median, IQR 0.7 km${}^{2}$, $N_{\mathrm{Parties}}=5$) and 23.0 km${}^{2}$ (median, IQR 2.3 km${}^{2}$, $N_{\mathrm{Parties}}=5$) in 2010, 2011, and 2012, respectively (Fig. 7). Home ranges (HRs) of baboon parties of the same gang and of different gangs overlapped on average by 88.1 % ± 9.3 % and 71.1 % ± 13.8 %, respectively (means ± SDs). The average minimum DTD of the Guinea baboons during the study period was 4010 m (IQR 2437 m). In each year, DTDs were relatively similar with 3998 m (IQR 2345 m), 4108 m (IQR 2471 m), and 3944 m (IQR 2502 m) in 2010, 2011, and 2012, respectively. But the longest minimum DTD reached 12.7 km, whereas the shortest was just 509 m (Fig. 8).

#### 3.2.3 Seasonality

For the entire study period, average home ranges of the Guinea baboons were smaller in the dry season (median: 19.1 km${}^{2}$, IQR 7.6 km${}^{2}$) than in the rainy season (median: 27.4 km${}^{2}$, IQR 6.6 km${}^{2}$), but with a reverse pattern in 2010. Similarly, average DTDs tended to be shorter in the dry season (3,465 m, IQR 2,213 m) than in the rainy season (4,428 m, IQR 2,356 m).

**Figure 9:**
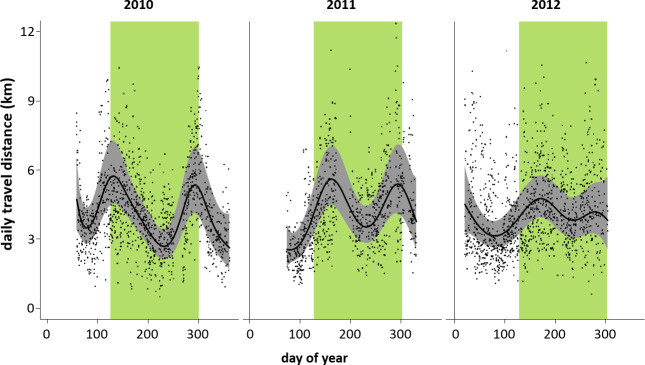
Minimum daily travel distances (DTD) of Guinea baboons in Simenti on a daily temporal scale. Grey dots represent individual DTD values, the black line is the smoothed mean travel path distances derived by the generalized additive model, and the grey shaded area represents the confidence bands at 99 %. The green bars indicate the rainy season (June to October).

DTDs fluctuated in the same way over 3 years (2010–2012) irrespective of individuals, parties, or gangs (Fig. 9). In each of the 3 years, there were two peaks (less pronounced in 2012): one at the beginning of the rainy season and one towards its end.

#### 3.2.4 Sleeping sites

**Figure 10:**
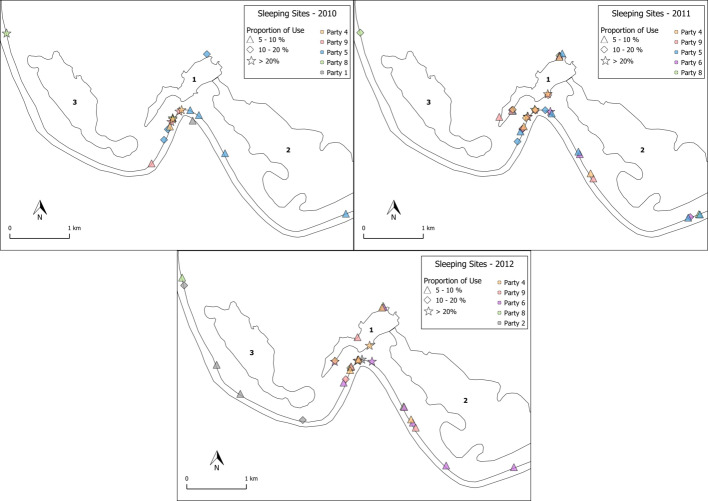
Distribution of main sleeping sites in 2010, 2011, and 2012. Star shapes depict locations that had been used in >20 % of all sleeping events. Diamond shapes represent 10 %–20 %, while triangles represent 5 %–10 % of all sleeping events. Sleeping locations <5 % are not depicted. Numbers depict the various wetland features: 1 – Mare Simenti, 2 – temporary wetland Simenti, 3 – temporary wetland Mare Kountadala. The two parallel lines represent the Gambia River.

The baboons spent the nights predominantly in their core areas (84 %, SD 7.5, range: 70.1 %–94.7 %) in the riverine forest close to the Gambia River or the local wetlands (Fig. 10). Dense vegetation and tall trees (>15 m) characterize these areas. Although the baboons spent the majority of nights in the riverine forest, they usually did not use the same cluster of trees as in the night before. On other evenings, they used tall trees near the temporary wetlands, and in rare cases, if they spent the day far away from the river, they also slept in trees out in the savannah. Important tree species used as sleeping sites by the baboons were palms (*Borassus akeassii*), kapok trees (*Ceiba pentandra*), African nettle trees (*Celtis integrifolia*), and the rosewood tree (*Pterocarpus erinaceus*). Certain trees appear to be especially suitable for protection against predators at night because they are difficult to climb (*Borassus akeassii*) or the bark is covered with large thorns (*Ceiba pentandra*).

#### 3.2.5 Habitat use

**Figure 11:**
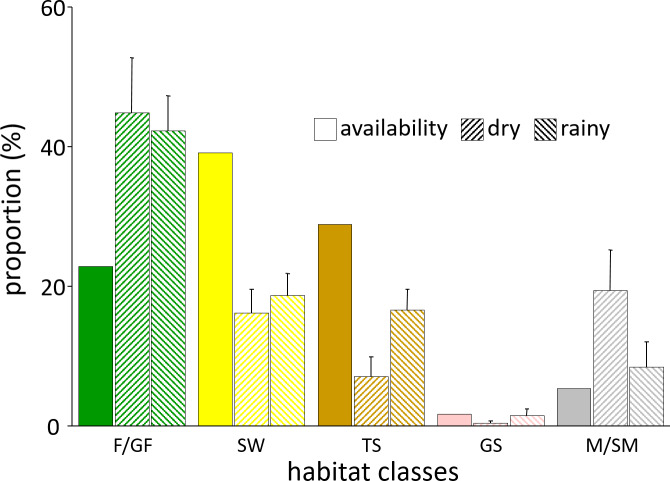
Habitat availability and habitat use of Guinea baboons in Simenti. Availability: proportion of habitat classes allocated within the area of interest. Use by baboons in dry and rainy season: proportion of location points of individual baboons within respective habitat classes in dry and rainy seasons. Individual variability in habitat use is displayed as the standard error. F/GF – forest/gallery forest; SW – savannah woodland; TS – tree savannah; GS – grassland; M/SM – Mare, seasonal wetland.

The most prevalent habitat type in the Simenti area was savannah woodland (SW), comprising approximately 39 % of the area of interest followed by the tree and shrub savannah (TS, 29 %), while forests (F/GF) covered approx. 23 % of the total area. Only 5 % of the area available to the baboons was identified as Mare/temporary wetlands (M/SM), and 2 % was covered by grass savannah (GS). The baboons used forest habitats and wetlands more frequently than a random distribution would suggest (Fig. 11). Despite their wide availability, the savannah habitats were underrepresented in the utilization pattern. Notably, these habitats show a more intense utilization pattern during the rainy season, in particular, the tree and bush savannah.

#### 3.2.6 Diet

Preliminary data suggest that Guinea baboons at Simenti have the opportunity to feed on a variety of woody plant species (i.e. trees, shrubs, and lianas). We observed feeding at least once per species from 53 woody plants belonging to 21 families out of a total species pool of >70 woody species. Hence, the baboons use a considerable portion of the occurring woody vegetation as a food resource. The most common food items consumed were fruits, either fleshy, indehiscent (pulp containing seeds), or dry (in)dehiscent fruit types (pods, samaras, capsules containing seeds). Dry fruits are mostly available in the dry season (e.g. *Bombax costatum*, *Pterocarpus erinaceus*, *Piliostigma *spp., and *Terminalia macroptera*), while the majority of fleshy fruits are restricted to the rainy season or shortly after (e.g. *Spondias mombin*, *Lepisanthes senegalensis*, *Tamarindus indica*, *Celtis integrifolia*; except *Strychnos spinosa*, *Lannea *spp.). The most important food item that is consumed by the baboons nearly year-round (i.e. staple) is the fruit of *Borassus akeassii*, which is abundant in the gallery forests close to the river and the wetlands. The *Borassus* fruits occur as food items in a variety of developmental stages, ranging from unripe to fully matured fruits (orange-yellow fibres), including the hard seeds. Baboons also frequently fed on a variety of herbaceous plants such as *Echinochloa *spp., *Chrysopogon* spp., and *Costus spectabilis*, including aquatic species (e.g. *Nymphaea lotus*).

#### 3.2.7 Nutritional value

The nutritional values of food items consumed by Guinea baboons fall within the range of the chemical composition of plant items consumed by yellow baboons in the moist gallery forest of Tana River and the adjacent dry savannah habitat, thus covering most of the range of habitats used by baboons (Table 1). The comparison is restricted to plant chemicals analysed by both studies in a comparable way. Some plant dietary items at Simenti were characterized by very high concentrations of energy-providing nutrients, such as ripe *Borassus* fruits containing more than 60 % sugar or other easily soluble and hydrolysable carbohydrates per dry weight. Soluble carbohydrate concentrations of 46 % occurred also in tubers of yam. Seeds of most Fabaceae contained around or above 20 % of crude protein. Leaves were consumed almost exclusively from herbs during the wet season, and flowers were consumed during the dry season, both food categories containing about 10 % of crude protein. Fat and secondary plant chemicals seem to play a minor role in the plant diet of the Guinea baboons. Alkaloids occurred in 9 out of 17 seeds and in 3 fruits, but not in any other food item (Table S1).

**Table 1:** Comparison of the chemical composition of vegetable food consumed by Guinea baboons (Pp) at CRP Simenti and by yellow baboons (Pc) in a forest and savannah habitat at the Tana River Primate National Reserve, Kenya (data from Bentley-Condit and Power, 2018). Values are **medians**, quartiles, and ranges of percentages based on dry matter.

	NDF	ADF	Nitrogen	Lipids	Ash
Pp	36.07/**47.41**/62.07	20.48/**31.77**/39.57	0.84/**1.39**/2.08	0.86/**1.59**/3.33	3.00/**4.00**/6.19
N=91	12.99 – 85.80	2.78 – 68.31	0.26 – 5.60	0.00 – 23.14	0.37 – 24.72
Pc forest	37.38/**44.86**/58.51	27.09/**35.69**/43.96	0.93/**1.25**/1.77	1.37/**4.56**/8.24	3.71/**4.91**/6.37
N=35	25.72 – 76.38	13.41 – 63.90	0.60 – 3.08	0.19 – 17.50	1.68 – 13.90
Pc savannah	34.44/**51.60**/63.37	18.20/**34.65**/44.13	1.19/**1.58**/2.27	1.02/**2.14**/6.23	4.07/**6.22**/7.97
N=27	16.16 – 72.20	4.81 – 51.98	0.72 – 4.18	0.19 – 21.40	2.51 – 22.84

NDF – neutral detergent fibre; ADF – acid detergent fibre.

#### 3.2.8 Predation

To date, no quantitative data on predator density and predation risk are available for the Simenti area. Based on field surveys of the PNNK management and opportunistic encounters, apex predators in the region are leopards (*Panthera pardus*) and lions (*Panthera leo*), but spotted hyenas (*Crocuta crocuta*) and African wild dogs (*Lycaon pictus*) have also been observed in the area (Ndao and Henschel, 2011, personal observation). Like other baboons, Guinea baboons also act as mesopredators hunting smaller vertebrates, in particular fawns of bushbuck (*Tragelaphus scriptus*), other ungulates, and hares (Goffe and Fischer, 2016).

## 4 Discussion

### 4.1 Interspecific comparison

Baboons in general are ecologically flexible and occur in various ecozones with a range of climate conditions and habitats. Although their specific distributions are correlated with climatic variables to some degree, their climatic niches overlap largely (Fuchs et al., 2018; Chala et al., 2019). For instance, Fuchs et al. (2018) found a strong overlap between the climate niches of Guinea with Kinda baboons, and Chala et al. (2019) found a strong overlap between Guinea and olive baboons. Baboon populations and species are probably “demographically interchangeable” in the sense of Templeton (1989).

The distribution range of Guinea baboons, although small compared to other baboon species, overlaps several ecozones from semi-desert, savannah, and woodland to tropical moist forest and mangroves. Not surprisingly, in their range we found the highest average annual precipitation, as well as the largest precipitation range and the highest average seasonality in annual precipitation of all baboon species. In the extreme, Guinea baboons live in habitats that are ecologically similar to the driest habitats of hamadryas and chacma baboons, as well as in habitats as humid as the most humid habitats of olive and Kinda baboons.

### 4.2 Local ecology of Simenti baboons

#### 4.2.1 Home range size and DTD

First of all, caution is needed when home range sizes are compared across studies, because HR size and DTD estimates heavily depend on estimation techniques and methods, in addition to ecological and demographic factors (e.g. Laver and Kelly, 2008; Pebsworth et al., 2012; Gula and Theuerkauf, 2013). Thus, such comparisons can only provide at some rough information on the magnitude of HR and DTD.

Home range size, daily travel path length, habitat use, and diet of Guinea baboons at Simenti fall within the range of *Papio* (Johnson et al., 2015; Swedell, 2011; Table 2). Home ranges varied across years and parties with an average of 24.8 km${}^{2}$ (range: 16.9–41.6 km${}^{2}$; KDE estimates) but tended to be larger in rainy seasons. Sharman (1981) provided some preliminary data on home range sizes of two Guinea baboon groups from the eastern part of the PNNK (20 and 42 km${}^{2}$), an area that is characterized by drier habitats (i.e. Mount Assirik) compared to Simenti. However, it is not clear if the estimates represent home range sizes on the party or gang level. For hamadryas baboons, Sigg and Stolba (1981) gave a range size of 28 km${}^{2}$ at Erer Gota, Ethiopia, while Boug et al. (1994) estimated an average annual home range size of 6.9 km${}^{2}$ in the Alhada Mountains of Saudi Arabia (monthly variation: 4.0–9.3 km${}^{2}$). The largest hamadryas home ranges were estimated for Filoha, Ethiopia, with ca. 40 km${}^{2}$ (Schreier, 2009), while a more recent GPS study suggests much larger home ranges (75.3 km${}^{2}$, Table 2; Henriquez et al., 2021). In the older literature, HR sizes are often given as estimates of MCPs, which tend to overestimate “true” HR sizes. For example, home range sizes in Guinea baboons were considerably larger when using the minimum convex polygon (MCP) method instead of KDE (KDE = 24.8; MCP up to 100 km${}^{2}$), making maximum HR sizes in Guinea baboons similar to the large home ranges reported from hamadryas baboons (MCP 129.3 km${}^{2}$ Henriquez et al., 2021).

**Table 2:** Home range size (HR) and daily travel distance (DTDs) of baboons. Depending on the study, these data represent single values, means, and/or ranges. Since the estimations of HR and DTD are based on different group sizes and since different methods were used, the values are only comparable to a limited extent.

Taxon	Site	HR km${}^{2}$	DTD km	Reference
Pu	Cape, ZAF	10.7 and 12.7	4.7 (1.6–8.0)	Hall (1962)
Pu	Cape, ZAF	37	7.9 (3.0–13.8)	Davidge (1978)
Pu	Cape, ZAF	11 (1.5–37.7)	4.0 (1.7–6.6)	Hoffman (2011)
Pu	DeHoop, ZAF	12.4 and 18.8		Hill (1999)
Pu	Drakensberg high, ZAF	18.9	4.3 (3.0–6.0)	Whiten et al. (1987)
Pu	Drakensberg low, ZAF	10	3.8 (2.7–5.0)	Whiten et al. (1987)
Pu	Hornett, ZAF	12.9–23.3	12.9 (3.9–23.3)	Stoltz and Saayman (1970)
Pu	Kuiseb, NAM	4.0 and 9.7		Hamilton et al. (1976)
Pu	Mkuzi, ZAF	24.4	4.9	Gaynor (1994)
Pu	Moremi, BWA	10		Bulger and Hamilton (1987)
Pu	Moremi, BWA	5 (flooded area)		Cheney et al. (2004)
Pu	Moremi, BWA	2.1–6.5		Hamilton et al. (1976)
Pu	Suikerbosrand, ZAF	13.7–22.4	3.1–5.4	Anderson (1981)
Pu	Tsaobis, NAM	12.3 and 26.8	6.0 (1.7–9.8)	King (2008)
Pu		1.5–37.7	1.6–23.3	
Pc	Amboseli, KEN	24	5.5 (4.6–6.0)	Altmann and Altmann (1970)
Pc	Amboseli, KEN		3.0–6.9	Bronikowski and Altmann (1996)
Pc	Amboseli, KEN	15.3 (5.6–24.8)	5.0 (3.5–8.3)	Markham (2012)
Pc	Amboseli, KEN	12.6–19.6		Stacey (1986)
Pc	Issa Valley, TZA	2.3 and 5.8	3.7–4.7	Johnson (2015)
Pc	Tana River, KEN		3.4–7.2	Wahungu (2001)
Pc		2.3–24.8	3.0–8.3	
Pa	Comoé, CIV	4.1 and 16.6		Kunz and Linsenmair (2008)
Pa	Gashaka Gumti, NGA	1.5	2.4 and 3.1	Warren et al. (2011)
Pa	Gilgil, KEN	19.7	4.6 (2.2–7.8)	Harding (1976)
Pa	Gombe, TZA	3.9–5.2	1.6–3.2	Ransom (1981)
Pa	Laikipia KEN	43.8	5.6	Barton et al. (1992)
Pa	Metahara, ETH	4.3	5.8	Aldrich-Blake et al. (1971)
Pa	Nairobi, KEN	25.8		DeVore and Washburn (1963)
Pa	Nairobi, KEN	23.2		DeVore and Hall (1965)
Pa	QENP, UGA	5.2 and 3.9	1.6–2.4 (max 6.4)	Rowell (1966)
Pa		1.5–43.8	1.6–7.8	
Ph	Erer Gota, ETH		13.2 (9.8–19.2)	Kummer (1968a)
Ph	Erer Gota, ETH	28.0	8.6 and 10.4	Sigg and Stolba (1981)
Ph	Filoha, ETH	30.0	7.5 (3.2–11.2)	Swedell (2002)
Ph	Filoha, ETH	38.6	8.3 (4.6–14.2)	Schreier (2009)
Ph	Filoha, ETH (95 % KDE)	75.3		Henriquez et al. (2021)
Ph	Filoha, ETH (100 % MCP)	129.3		Henriquez et al. (2021)
Ph	Taif, SAU	6.9 (4.0–9.3)	1.0–14.0	Boug et al. (1994)
Ph		4.0–129.3	1.0–19.2	
Pp	Mt. Assirik, SEN	20.0 and 42.0	4.0–13.0	Sharman (1981)
Pp	Simenti, SEN (95 % KDE)	24.8 (per party)	4.0 (0.5–12.7)	this study
Pp	Simenti, SEN (100 % MCP)	45 (per party) up to 100		this study
Pp		20.0-100.0	0.5-13.0	

BWA – Botswana, CIV – Ivory Coast, ETH – Ethiopia, KEN – Kenya, NAM – Namibia, NGA – Nigeria, TZA – Tanzania, SAU – Saudi Arabia, UGA – Uganda, ZAF – South Africa, QENP – Queen Elizabeth National Park.

Similar problems occur when DTDs are compared among different studies. The distance estimate largely depends on the number of geographical positions available per daily march, because it is often not possible to record the travel path continuously. The DTDs presented in Figs. 8 and 9 are based on GPS fixes taken every 2 h; thus, they represent minimum distances covered by the respective baboons. The average underestimation of the true DTD is >25 % (Sennhenn-Reulen et al., 2017). Adding the 25 % to our estimated DTDs makes the maximum DTDs of the Simenti baboon similar to those of hamadryas DTDs (Filoha, Ethiopia: 14.2 km (Schreier, 2009); Erer Gota, Ethiopia: 19.2 km (Kummer, 1968a); Taif, Saudi Arabia: 14 km (Boug et al., 1994)).

#### 4.2.2 Habitat use and sleeping sites

Sharman (1981) and Galat-Luong et al. (2006) provided some data on Guinea baboon habitat use. Since quantitative data on habitat availability were not available at that time, habitat preferences could not be determined. Therefore, we cannot directly compare our findings of habitat preference with the data of the previous studies. In both previous studies, usage of habitat types was very similar, with shrubby savannahs showing the highest utilization, followed by arboreal savannah, forests, and open grassland. In our study, in contrast, the baboons preferred the forest habitats, mainly along the river and around the wetlands. Aside from food availability, this preference is probably also linked to the availability of water sources and tall trees used as sleeping sites. Sharman (1981) also reported that baboons used riverine forests as sleeping sites, similar to the majority of cases of our study population. However, no permanent water source was present in his study area. Therefore, the area covered by forests was potentially smaller than at the Simenti field site. Furthermore, Sharman's study took place in the eastern part of the PNNK, which is characterized by different topsoil formations and elevation regimes that might lead to a very different vegetation structure and distribution (Dupuy, 1971; Hejcmanova-Nežerková and Hejcman, 2006).

#### 4.2.3 Population density

The estimated population density in the area around Simenti of 7.5–10 baboons per square kilometre is slightly higher than the estimates by Galat et al. (2009) for the entire PNNK (1990–1998: 6.3–7.3 baboons per square kilometre). This might result from the availability of permanent water sources at Simenti and thus a relatively productive habitat, compared to other parts of the national park. Population densities of hamadryas baboons, with their similar social organization, are generally lower (1.8 baboons per square kilometre in Erer Gota, Ethiopia (Kummer, 1968a), and 3.4 baboons per square kilometre in Awash, Ethiopia (Nagel, 1971)). But densities can exceed these levels in highly productive landscapes. For two areas in Eritrea, Zinner et al. (2001) estimated densities of 10.2 and 23.9 baboons per square kilometre respectively. Particularly the latter area was among the most productive areas in the hamadryas baboon range of Eritrea, covered with prickly pear, *Opuntia ficus-indica*, which provided year-round food.

#### 4.2.4 Seasonality and diet

Although we detected differences between dry and rainy season in Guinea baboons at Simenti with larger HRs and longer DTDs in the rainy season, this dichotomic categorization does not seem to be justified for an appropriate categorization of the seasonal patterns and subsequently the ranging patterns. Instead, oscillating patterns of DTDs on finer temporal scales suggest similar maxima and minima over the year independent of dry and rainy seasons. Seasonal variation in resource availability (i.e. phenological patterns) and preferred foods between different habitat types of the forest–savannah mosaic likely account for the variance in DTDs at Simenti. Sharman (1981) did not detect any seasonal differences in DTDs but noted great daily variation in travel patterns. Preliminary information on feeding and phenology suggests that the baboons at Simenti have the opportunity to forage on a variety of woody plants in savannah habitats (>70 species) that bear fleshy fruit outside the rainy season, among others *Cordyla pinnata*, *Ficus ingens*, *Sclerocarya birrea*, and *Strychnos spinosa*. Moreover, woody plant species with pods and samaras containing seeds that are rich in protein (Table S1) are largely exploitable in savannah habitats outside the rainy season, among others, *Acacia seyal*, *Bombax costatum*, *Combretum spp*., *Piliostigma *spp., *Pterocarpus erinaceus*, and *Terminalia macroptera*. Similar to hamadryas baboons at Filoha, Guinea baboons at Simenti have the opportunity to feed on palm fruits (*Borassus akeassii*), which constitute a major food resource and are consumed at various stages of development. In particular, when ripe, the fibrous orange fruit contains high amounts of sugar (Table S1). Preliminary phenological observations suggest that Guinea baboons at Simenti tend to show minima in DTDs during times of fruit availability: once in mid-rainy season and once in mid-dry season. The increase in DTDs shortly after might be related to the depletion of resource patches close to the sleeping sites and the expansion in range use to seek out less visited areas and/or new food resources.

## 5 Conclusion

Guinea baboons occur under a considerable range of ecological and climatic conditions, and within this range, the conditions of our study population do not represent extremes. It was hypothesized that the multi-level social organization of hamadryas baboons is an adaptation to the harsh ecological conditions of their arid semi-desert habitat with its specific distribution of food resources and safe sleeping cliffs and relatively low predation pressure (Kummer, 1968b, 1990; Dunbar, 1988; Barton, 2000). The majority of the Guinea baboon populations live under considerably different ecological condition than hamadryas baboons, yet Guinea baboons show a similar social organization as hamadryas baboons (Boese, 1975; Fischer et al., 2017). Despite pronounced differences in the habitats of hamadryas and Guinea baboons, it appears that the multi-level social organization of both species is functional in different habitats. An interesting next step would be a detailed quantitative comparison of the social and sexual relationships of the animals, preferably using the same sampling and analytical protocols (as in Kalbitzer et al., 2015). Moreover, it would be highly desirable to conduct a socio-ecological study of Guinea baboons in their northern, semi-desert range in Mauretania, where the ecological situation might be more similar to the habitats of hamadryas baboons. Such data would be crucial for further comparisons with hamadryas baboons and a deeper understanding of the adaptive value of the multi-level social organization in baboons.

## Supplement

10.5194/pb-8-19-2021-supplement The supplement related to this article is available online at: https://doi.org/10.5194/pb-8-19-2021-supplement.

## Data Availability

Raw data of the chemical analysis of food items are provided in Table S1. GPS data for the estimation of HR and DTD can be found on Göttingen Research Online: (Zinner et al., 2021).
