# Supplementary material for: Comparative ecology of Guinea baboons (Papio papio)
Source: Primate Biol. 2021 May 21;8(1):19–35. doi: 10.5194/pb-8-19-2021 (PMC8182668; doi:10.5194/pb-8-19-2021)
Supplement: The supplement related to this article is available online at: https://doi.org/10.5194/pb-8-19-2021-supplement. [file pb-8-19-supplement.pdf]

Supplement of Primate Biol., 8, 19–35, 2021
https://doi.org/10.5194/pb-8-19-2021-supplement
© Author(s) 2021. CC BY 4.0 License.

Primate Biology Open Access

Supplement of

Comparative ecology of Guinea baboons (*Papio papio*)

Dietmar Zinner et al.

Correspondence to: Dietmar Zinner (dzinner@gwdg.de, dzinner@dpz.eu)

The copyright of individual parts of the supplement might differ from the article licence.

Supplement

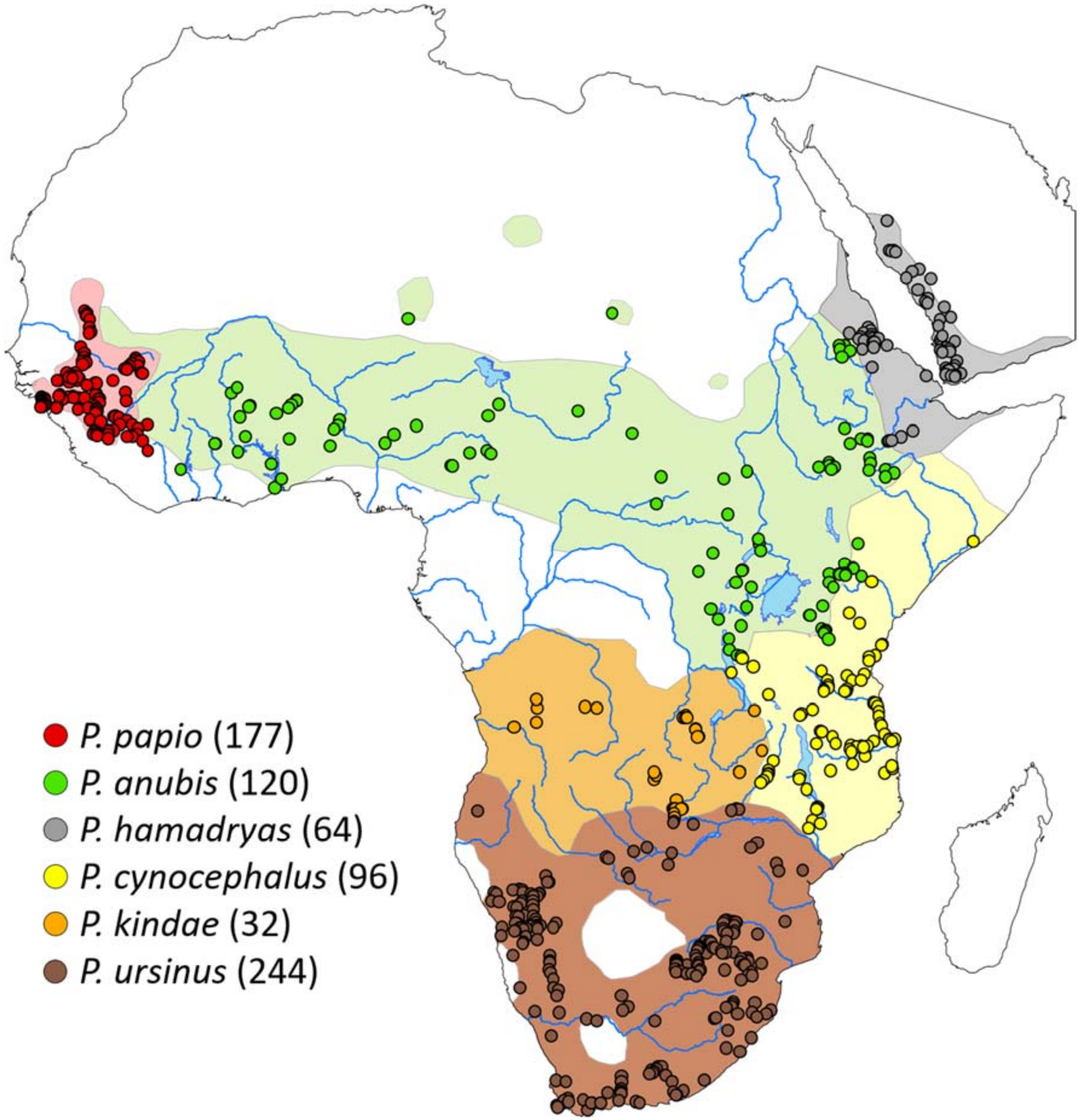

Figure S1: Approximate distribution of the six baboon (*Papio*) species and geographic positions of occurrence points (redrawn from Chala et al. 2019).

Table S1: Chemical components of food items consumed by *Papio papio* at Simenti collected during the wet and the dry season. Values are percentages of dry weight, except for alkaloids that have been analysed only qualitatively; pos = positive, neg = negative.

Species	Family	Plant Item	Nitrogen	ADF	NDF	Sugar	cond. Tannins	Poly-phenoles	Lipids	Ash	Alkaloid Dragen-dorff	Alkaloid Mayer	Alkaloid Wagner
Acacia macrostachya	Fabaceae (Mimosoidae)	seeds	4.76	16.67	25.67	3.56	0.12	0.45	4.30	2.94	pos	pos	pos
Acacia seyal	Fabaceae (Mimosoidae)	fruits pods; including seeds	2.36	22.66	32.78	7.59	0.44	6.32	0.59	4.01			
Acacia spec.	Fabaceae (Mimosoidae)	bark	1.19	39.57	52.32	9.32	2.73	3.27	0.83	5.24	neg	neg	neg
Acacia spec.	Fabaceae (Mimosoidae)	fruits	2.21	24.53	41.71	13.60	2.44	4.35	1.31	4.52	neg	neg	neg
Afzelia africana	Fabaceae (Caesalpinioideae)	seeds	2.90	19.36	43.99	6.24	0.14	0.38	14.63	2.08	pos	neg	pos
Anogeissus leiocarpus	Combretaceae	fruits	0.71	38.92	64.54	5.95	0.20	3.35	0.00	1.96	neg	neg	neg
Aphania senegalensis (Lepisanthes senegalensis)	Sapindaceae	fruits including seeds	1.15	25.58	43.51	12.07	0.00	0.24	2.87	3.04			
Bombax costatum	Malvaceae (Bombacaceae)	seeds	3.84	29.69	47.41	2.41	0.19	0.28	6.97	4.03	pos	pos	pos
Borassus akeassii	Arecaceae	fiber from stem	0.40	56.08	72.31	0.46	0.00	0.16	0.00	12.74	neg	neg	neg
Borassus akeassii	Arecaceae	fruit fiber	0.48	13.92	21.48	74.35	0.00	0.12	1.37	1.86	pos	neg	neg
Borassus akeassii	Arecaceae	fiber from leave	0.52	67.44	85.80	0.48	0.00	0.23	0.65	4.40	neg	neg	neg
Borassus akeassii	Arecaceae	fruitflesh from nut hart	0.72	54.58	75.55	5.87	0.00	0.05	0.54	0.37	neg	neg	neg
Borassus akeassii	Arecaceae	fiber from stem	0.84	41.70	85.10	0.74	0.00	0.24	0.87	9.72	neg	neg	neg
Borassus akeassii	Arecaceae	fruit	0.87	13.92	35.73	48.63	0.00	0.15	1.09	2.89			
Borassus akeassii	Arecaceae	young leaves	0.98	48.68	77.74	4.28	0.00	0.25	1.37	3.19	neg	neg	neg
Capparis tomentosa	Capparaceae	fruits	2.93	20.45	40.37	5.83	0.15	0.45	4.85	5.13	pos	pos	pos
Cassia sieberiana	Fabaceae (Caesalpinioideae)	seeds	2.30	10.46	38.59	9.31	0.97	0.93	0.62	3.47	pos	pos	pos
Celtis integrifolia	Cannabaceae	fruits	2.14	21.45	46.08	2.93	0.98	0.60	1.88	24.72			
Cissus populnea	Vitaceae	fruits	1.34	30.40	52.50	6.69	0.39	0.40	12.89	9.33	neg	neg	neg
Combretum glutinosum	Combretaceae	fruits samara; including seeds	1.98	27.35	42.08	9.04	0.00	6.38	14.73	3.14	pos	neg	neg
Combretum micranthum	Combretaceae	fruits	1.67	33.12	48.90	10.93	2.99	3.95	3.57	3.41			
Combretum nigricans	Combretaceae	fruits samara; including seeds	1.18	40.15	56.38	6.00	1.24	3.21	0.63	3.02	pos	neg	neg
Cordyla pinnata	Fabaceae (Caesalpinioideae)	fruits without seeds	1.08	7.29	12.99	25.51	0.56	1.50	1.37	3.23	neg	neg	neg
Cordyla pinnata	Fabaceae (Caesalpinioideae)	seeds	1.99	2.78	25.37	16.68	0.00	0.09	1.16	2.69			
Cordyla pinnata	Fabaceae (Caesalpinioideae)	flowers	2.08	11.70	19.97	26.04	0.84	1.44	1.92	4.64	neg	neg	neg
Crossopterix febrifuga	Rubiaceae	seeds	1.00	68.31	77.58	1.62	0.12	0.60	0.00	2.46	neg	neg	neg
Detarium microcarpum	Fabaceae (Caesalpinioideae)	fruits including seeds	0.61	40.69	59.01	6.12	1.22	2.46	2.07	1.55	neg	neg	neg

Dichrostachys cinerea	Fabaceae (Mimosoidae)	fruits pods; including seeds	1.87	22.38	35.93	15.82	3.30	5.21	0.00	5.64	neg	neg	neg
Diospyros mespiliformis	Ebenaceae	fruits including seeds	0.69	38.73	56.69	9.49	0.79	2.29	1.81	2.90			
Dombeya quinqueseta	Malvaceae (Sterculiaceae)	flowers	1.75	23.35	38.92	15.54	2.05	2.97	1.29	6.37	pos	neg	neg
Ficus ingens	Moraceae	ripe fruits	0.66	34.97	47.57	11.50	1.18	1.61	2.99	5.93	neg	neg	neg
Ficus ingens	Moraceae	unripe fruits	0.67	32.05	47.61	8.47	0.94	0.73	3.22	6.48	neg	neg	neg
Ficus iteophylla (thonningii)	Moraceae	fruits including seeds	0.80	32.31	45.78	8.18	1.28	2.04	2.56	7.46			
Grewia lasiodiscus	Tiliaceae	seeds	0.61	59.22	78.47	3.45	0.00	0.21	0.89	3.21	neg	neg	neg
Grewia lasiodiscus	Tiliaceae	fruits including seeds	0.86	33.53	45.93	6.99	0.97	1.26	4.13	8.18	neg	neg	neg
Grewia spec.	Tiliaceae	fruits	0.93	44.78	61.40	5.01	0.90	1.32	1.41	5.01			
herb			0.26	48.99	73.84	12.31	0.00	0.20	0.62	3.48	neg	neg	neg
herb			0.81	41.84	69.42	1.83	0.00	0.63	2.04	12.49	neg	neg	neg
herb			1.16	46.06	74.54	0.87	0.00	0.15	1.90	8.80	neg	neg	neg
herb			1.25	35.86	62.07	2.46	0.00	0.61	3.19	9.50	neg	neg	neg
herb			1.26	33.09	57.33	1.27	0.00	0.30	2.96	11.54	neg	neg	neg
herb			1.39	35.70	67.56	2.93	0.00	0.66	1.01	6.13	neg	neg	neg
herb			1.71	30.48	65.16	2.14	0.00	0.76	1.28	11.03	neg	neg	neg
herb			1.75	30.09	62.73	5.94	0.00	0.62	1.29	12.50	neg	neg	neg
herb			2.05	38.98	69.74	2.60	0.00	0.26	1.71	8.58	neg	neg	neg
herb			2.05	30.63	70.52	4.72	0.00	0.69	2.12	4.31	neg	neg	neg
herb		fruits pods	2.16	35.81	56.02	5.31	0.53	0.80	0.42	3.49	neg	neg	neg
herb	Laminaceae, big leaves		2.52	35.86	48.77	2.73	0.00	2.35	3.41	10.13	neg	neg	neg
herb	Laminaceae, small leaves		2.98	32.82	44.32	3.06	0.00	2.52	3.75	8.65	neg	neg	neg
herb			3.27	15.68	43.08	4.87	0.24	0.87	3.33	12.85	neg	neg	neg
Hexalobus monopetalus	Annonaceae	ripe fruits	0.99	28.63	43.45	25.14	0.49	0.40	6.07	3.77	neg	neg	neg
Hexalobus monopetalus	Annonaceae	unripe fruits	1.44	47.58	63.74	4.01	0.74	0.59	6.22	3.06	neg	neg	neg
Hexalobus monopetalus	Annonaceae	leaves	2.08	25.26	45.79	10.29	2.60	2.89	2.57	7.26	neg	neg	neg
Hexalobus monopetalus	Annonaceae	flowers	2.49	20.48	30.75	11.71	1.29	2.56	2.65	4.67			
Lannea microcarpa	Anacardiaceae	fruits including seeds	1.57	29.78	45.74	3.88	0.28	1.55	4.46	4.84			
Lannea velutina	Anacardiaceae	fruits including seeds	1.43	27.02	39.37	4.39	0.59	2.62	1.62	3.20	neg	neg	neg
Lonchocarpus laxiflorus	Fabaceae (Papilionoideae)	seeds	4.83	13.83	21.61	10.13	0.26	0.77	15.36	3.36	pos	pos	pos
Maytenus senegalensis	Celastraceae	fruits including seeds	1.24	18.27	36.07	7.01	1.64	2.49	23.14	4.67	pos	neg	neg
Mimosa pigra	Fabaceae (Mimosoidae)	fruits pods; including seeds	1.60	33.02	50.27	4.58	0.72	2.45	0.56	3.57	pos	neg	neg
Mimosa pigra	Fabaceae (Mimosoidae)	fruits pods; including seeds	1.71	38.00	49.32	2.44	0.80	1.92	0.61	4.31	neg	neg	neg
parasite			1.82	21.05	27.91	6.73	1.84	7.74	0.85	4.36	neg	neg	neg
Parkia biglobosa	Fabaceae (Mimosoidae)	fruit shell	1.42	21.73	30.87	18.16	5.46	7.68	0.19	3.39	neg	neg	neg
Parkia biglobosa	Fabaceae (Mimosoidae)	flowers	2.00	16.84	26.39	19.81	1.07	3.30	0.80	4.64	pos	neg	neg
Parkia biglobosa	Fabaceae (Mimosoidae)	seeds	3.03	12.84	20.95	7.85	1.34	3.58	1.66	4.01			
Piliostigma reticulatum	Fabaceae (Caesalpinioideae)	seeds	3.55	15.30	36.38	7.60	1.32	1.81	1.57	2.46	neg	neg	neg
Piliostigma spec.	Fabaceae (Caesalpinioideae)	old pods	0.32	53.97	65.84	0.13	0.00	0.21	0.51	2.22	neg	neg	neg

Piliostigma thonningii	Fabaceae (Caesalpinioideae)	seeds	3.42	13.15	33.45	9.43	1.51	1.58	1.04	2.72	neg	neg	neg
Pterocarpus erinaceus	Fabaceae (Papilionoideae)	bark	1.72	21.17	34.45	10.88	2.63	3.03	0.19	5.39	neg	neg	neg
Pterocarpus erinaceus	Fabaceae (Papilionoideae)	seeds	5.60	15.42	23.59	9.97	0.20	1.66	15.83	3.60	pos	pos	pos
Pterocarpus lucens	Fabaceae (Papilionoideae)	fruits pods including seeds	1.29	44.23	59.36	4.35	0.59	0.76	0.20	3.99	neg	neg	neg
Pterocarpus santaloides	Fabaceae (Papilionoideae)	fruits including seeds	2.27	26.21	47.65	7.31	1.29	1.47	2.86	2.93	pos	neg	pos
Saba senegalensis	Apocynaceae	seeds	1.02	34.19	55.06	7.30	0.00	0.18	4.46	2.94	neg	neg	neg
Sarcocephalus latifolius	Rubiaceae	fruit	1.02	33.79	42.04	5.81	1.21	1.68	4.68	5.57			
Sarcocephalus latifolius	Rubiaceae	flowers	1.83	17.11	26.05	5.52	0.12	1.70	1.33	6.82	neg	neg	neg
Sclerocarya birrea	Anarcadiaceae	fruits including seeds	0.80	37.82	46.78	15.15	0.29	0.50	7.32	3.45	neg	neg	neg
Sclerocarya birrea	Anarcadiaceae	seeds	0.91	48.19	63.94	5.60	0.00	0.27	4.11	0.79	pos	pos	pos
Senna siamea	Fabaceae (Caesalpinioideae)	seeds	3.38	14.55	34.94	5.76	0.00	0.13	3.77	4.24	pos	pos	pos
Spondias mombin	Anacardiaceae	fruits	0.74	45.04	53.52	7.52	0.12	0.51	2.43	3.65	neg	neg	neg
Strychnos spinosa	Loganiaceae	fruits shell	0.32	42.24	62.43	27.70	0.00	0.38	0.40	0.81	neg	neg	neg
Strychnos spinosa	Loganiaceae	fruits	0.94	32.00	44.61	16.18	0.00	0.75	1.31	3.08	neg	neg	neg
Tamarindus indica	Fabaceae (Caesalpinioideae)	shell	0.59	45.63	62.65	6.49	0.99	1.26	0.89	1.62	neg	neg	neg
Tamarindus indica	Fabaceae (Caesalpinioideae)	shell content including seeds	1.52	14.10	21.73	13.18	1.65	1.53	1.39	2.35	neg	neg	neg
Terminalia avicennioides	Combretaceae	flowers	1.00	27.86	37.08	10.02	0.42	5.84	1.24	4.12	neg	neg	neg
Terminalia macroptera	Combretaceae	seeds	0.72	51.90	68.26	3.07	0.00	1.95	1.21	1.46	neg	neg	neg
Terminalia macroptera	Combretaceae	flowers	1.51	16.59	23.33	12.33	0.90	4.06	3.15	7.34	pos	neg	neg
Vitex madiensis	Lamiaceae (Verbenaceae)	fruits	0.65	44.13	54.24	7.43	0.36	2.75	1.59	2.87	neg	neg	neg
Yams	Dioscoraceae		0.51	30.80	36.08	45.59	0.00	0.13	0.28	13.60	neg	neg	neg
Ziziphus mauritiana	Rhamnaceae	fruits including seeds	0.63	33.88	50.44	22.62	0.69	0.83	2.41	3.46	neg	neg	neg
Ziziphus mucronata	Rhamnaceae	fruits including seeds	0.85	36.84	50.92	22.55	1.38	1.68	1.59	2.84	neg	neg	neg
Ziziphus spec.	Rhamnaceae	fruits	1.78	31.77	50.66	5.38	0.59	1.54	0.86	#NULL!	neg	neg	neg
unidentified		fruits without shell	2.10	15.17	20.32	27.43	0.00	0.15	5.94	3.34	pos	pos	pos
